# A case for inclusion of “Taste Modification” in hypertension

**DOI:** 10.3389/fmed.2023.1096067

**Published:** 2023-06-02

**Authors:** Sudip Bhattacharya, Pratima Gupta, Saurabh Varshney, Amarjeet Singh

**Affiliations:** ^1^Department of Community and Family Medicine, All India Institute of Medical Sciences, Deoghar, India; ^2^Department of Microbiology, All India Institute of Medical Sciences, Deoghar, India; ^3^Department of ENT (Otorhinolaryngology), All India Institute of Medical Sciences, Deoghar, India; ^4^Department of Community Medicine, Post Graduate Institute of Medical Education and Research (PGIMER), Chandigarh, India

**Keywords:** Taste Modification, hypertension, non-communicable disease (NCD), epidemiologic transition, diabetes, prevalence, global burden disease, salt

Overconsumption of dietary sodium is linked to cardiovascular disease (CVD) risk factors, most notably increased blood pressure (BP). A significant risk factor for CVD, high blood pressure (HTN) kills 7.6 million people yearly, or 13.5% of all fatalities, worldwide. According to recent estimates, HTN is responsible for 47% of all coronary artery disease (CAD) and 54% of all strokes worldwide. Currently, the majority of HTN patients reside in low- and middle-income nations. Epidemiological research shows that HTN is becoming more common in both urban and rural India. According to estimates, there are 33% more people with HTN in urban regions than in rural ones in India. According to estimates, 57% of all stroke deaths and 24% of all CAD deaths in India are directly attributable to HTN. In 2010, sodium intake of more than 2 grams per day was linked to 1.65 million cardiovascular deaths worldwide. Various studies like INTERSALT have demonstrated that 17–30 percent of hypertension is caused by excessive dietary sodium consumption (1). Accordingly, over last 50 years or so, various studies have included salt reduction in their intervention package. But worthwhile results have failed to take roots at population level. Yet only an individual medical advice to reduce salt intake has been attempted for this goal without mentioning how it can be achieved by the patient alone. At family level, preparing/consuming food with low salt for an individual is often not practically feasible. Additionally, adapting with low salt diet requires a definite behavior change, which is a difficult process (2–5). Instead of just theoretically prescribing patients to lower their salt intake, we must provide an alternative practical regime by fostering a congenial environment that facilitates this behavior change.

We propose a “Taste Modification” concept for this purpose by utilizing two approaches. The long-term strategy involves preconditioning the infants to a low-salt diet from the weaning stage. If properly inculcated, this habit may be passed down through generations, as their taste buds may be physiologically conditioned to prefer low-salt diets.

Developing a taste or fondness for a food is a consequence of familiarity, so the things that our mothers eat, even before our birth, affects the way we'll respond to those flavors when we later encounter them because they seem familiar. So, early infancy is the most crucial time for the development of a person's eating preferences.

Most of the animal models and neuroimaging in humans suggested that the food can be used as a reward. Food intake can broadly be divided into two categories—homeostatic and hedonic. Homeostatic food intake is related to the basic need for food for the functioning of the body and hedonic food intake is related to getting a reward. In hedonic food intake, the person commonly takes fat-rich, sweet, or salty food for reward (6). When food stimulates the taste bud, the afferent nerve projects into the brainstem and is divided into two distinct pathways—a thalamic branch and a limbic branch. Hence, a significant input goes from the limbic areas to the gustatory cortex. This hedonic food intake is influenced by the central nucleus of the amygdala and is concerned about the appetite behavior and reward (7).

As an example, Infants may initially reject healthful foods like vegetables due to their intrinsic dislike of sour and bitter flavors. Children's dietary preferences and sensory experiences are influenced by parental feeding habits and environmental circumstances when solid foods are introduced through toddlerhood. Healthy eating habits among mothers can help children start out on the right foot as breastfed babies are more responsive to flavor which are transferred from the mother's food to the amniotic fluid and mother's milk (6). Infants can learn through repeated exposure and dietary variety, regardless of their early feeding style. Offering supplementary foods that are minimal in sugar and salt may help shield the developing youngster from excessive consumption in the future. Early exposure to nutritious tastes and flavors may help promote healthy eating habits, which may help to significantly lower the occurrence of the various chronic diseases connected to unhealthy food choices (6).

The short-term strategy is for the adults. In a number of trials, it was found that after a specific length of salt depletion from the diet, foods that were previously regarded normal were deemed salty (7). For instance, if we adopt the practice of consuming salads with less salt and more citric juice, our taste buds may behave differently with time. After some time, we no longer accept a salad with a high salt content if it is offered to us. Our taste senses would have been conditioned for this reason (8).

We know that, rather than being established by genetics, taste is primarily acquired *via* experience (9–12). Hence, it is easily manipulable. According to a Chinese study, adding herbs and spices can make previously unpalatable low-salt tomato soup more appealing to consumers. Customers first objected when the customary tomato soup's salt content was reduced and herbs and spices were added. But after some time, the same clients began to tolerate it well as a result of frequent exposure. When exposed to context-specific cues in the future, associative learning leads a basic action to become activated (that is, habitually), according to research. Once action initiation is “transferred” to external cues, dependence on conscious attention or motivational processes is reduced (13, 14).

In Southeast Asia, “Taste Modification” (Figure 1) among adults could represent a novel strategy to prevent or control hypertension. We have numerous possibilities for promotion of low-salt diet by modifying the flavor of food with masala (spices like turmeric, pepper, ginger). In hospital settings, adult hypertensive patients may be advised that adding spices/herbs such as ginger, black pepper, etc. to their food can be one good alternative to salt. The goal is to progressively substitute salt by these spices and minimize the risk of hypertension. Additionally, although it is far from fully understood, there is a well-established link between consuming sodium chloride and the development of hypertension. Blood pressure changes have also been linked to other taste categories including bitter, umami, or sweet. Here, we examine the connection between flavor and hypertension to uncover potential strategies for better blood pressure management. With the exception of a section on molecular pathways, the focus of this review is on published research involving people. There is strong evidence to support the idea that changes in the sensitivity to salt can be used to forecast when hypertension will start to develop. This is related to the medical idea of increased salt sensitivity with age, especially in hypertension patients. The identification of taste receptors (TAS1R and TAS2R) in the heart may change future pharmacological strategies to prevent heart diseases (15). In addition to biological variables, governmental and community-level influences may have an impact on people's decisions regarding breastfeeding and adult eating. The establishment of good food choices at a young age, which has the potential to convert into a healthy diet for life, requires a multi-layered strategy. “Taste Modification” approach may be further investigated by multicentric trials and if the level of scientific evidence found to be strong enough, it can be further incorporated in hypertension prevention guidelines.

**Figure 1:**
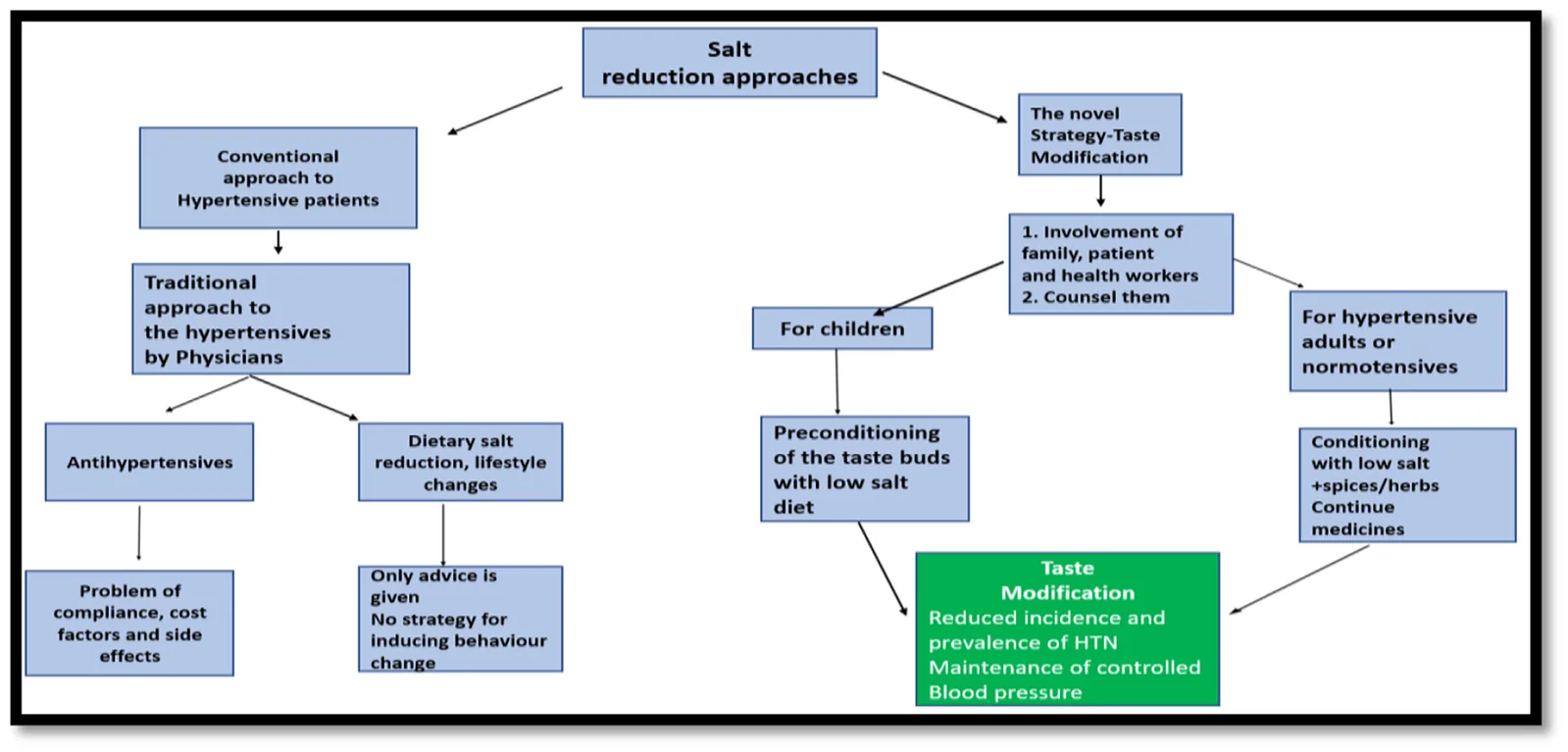
Salt reduction strategies. The schematic is an independently author-developed representation of the “Taste Modification” framework, based on the conceptual elements previously presented in the authors' 2016 publication on dietary salt reduction (17). A closely related schematic was subsequently developed and published by the authors in the Lancet Regional Health - Southeast Asia Viewpoint (16). The two schematics therefore share conceptual and certain structural elements because they derive from the same underlying author-developed framework.

## Author contributions

All authors listed have made a substantial, direct, and intellectual contribution to the work and approved it for publication.
