# Correction: A case for inclusion of “Taste Modification” in hypertension

**DOI:** 10.3389/fmed.2026.1977251

**Published:** 2026-10-30

**Authors:** Sudip Bhattacharya, Pratima Gupta, Saurabh Varshney, Amarjeet Singh

**Affiliations:** 1 Department of Community and Family Medicine, All India Institute of Medical Sciences, Deoghar, India; 2 Department of Microbiology, All India Institute of Medical Sciences, Deoghar, India; 3 Department of ENT (Otorhinolaryngology), All India Institute of Medical Sciences, Deoghar, India; 4 Department of Community Medicine, Post Graduate Institute of Medical Education and Research (PGIMER), Chandigarh, India

**Keywords:** Taste Modification, hypertension, non-communicable disease (NCD), epidemiologic transition, diabetes, prevalence, global burden disease, salt

In the published article, the relationship of this work to a closely related 2023 publication and to the authors' earlier publication introducing the “Taste Modification” concept was not stated explicitly. In addition, the provenance and relationship of Figure 1 to the corresponding schematic in the related publication were not sufficiently described in the original figure legend.

To ensure complete transparency of the scientific record, the following corrections and clarifications are provided.

The acknowledgement statement has been amended from

“We do acknowledge Dr. Himel Mondal, Assistant Professor, Department of Physiology, AIIMS, Deoghar for his expert input in this article. We do also acknowledge all the authors of those books, articles, and journals that were referred in preparing this manuscript.”

to read as follows:

“We acknowledge Dr. Himel Mondal, Assistant Professor, Department of Physiology, AIIMS, Deoghar, for his expert input in this article. A closely related paper was published by Bhattacharya et al. in *The Lancet Regional Health – Southeast Asia* in 2023 [16]. Both papers share the same underlying “Taste Modification” concept but differ in emphasis and purpose. The present *Frontiers in Medicine* article discusses the concept from a broader preventive and public-health perspective, including long-term taste conditioning from early life and a short-term strategy for adults, whereas the *Lancet Regional Health – Southeast Asia* Viewpoint focuses specifically on the application of taste modification to hypertension prevention and control in India and emphasizes the need for multicentric clinical trials before wider implementation [16]. Figure 1 in this article was developed by the authors from the framework described in [17]. A closely related schematic appears in [16].”

The legend of figure 1 has been corrected. *Lancet Regional Health – Southeast Asia* publication “16” was not cited in the article. The citation of the *Lancet Regional Health – Southeast Asia* publication [16] in this corrected legend is therefore intended to transparently disclose the closely related publication and the shared conceptual framework and does not indicate reproduction or adaptation of the Lancet figure.

The following citations have now been inserted in the legend of Figure 1 and should read:.

**“**“**Figure 1. Salt reduction strategies.** The schematic is an **independently author-developed representation** of the “Taste Modification” framework, based on the conceptual elements previously presented in the authors’ 2016 publication on dietary salt reduction [17]. A closely related schematic was subsequently developed and published by the authors in the *Lancet Regional Health – Southeast Asia* Viewpoint [16]. The two schematics therefore share conceptual and certain structural elements because they derive from the same underlying author-developed framework.**”**

**Additional references**

**16.** Bhattacharya S, Varshney S, Hossain MM, Saleem SM, Gupta P, Singh A. “Taste modification” strategy for prevention and control of hypertension in India: need for robust clinical trials. *Lancet Reg Health Southeast Asia.* 2023;14:100206. doi:10.1016/j.lansea.2023.100206.

**17.** Bhattacharya S, Singh A. “Taste Modification”–A New Strategy for Reduction of Salt Intake among Indian Population. *Epidemiol Int.* 2016;1(2):44–50.

The original version of this article has been updated.

